# A new portable toluidine blue/aptamer complex-on-polyethyleneimine-coated gold nanoparticles-based sensor for label-free electrochemical detection of alpha-fetoprotein

**DOI:** 10.3389/fbioe.2023.1182880

**Published:** 2023-05-22

**Authors:** Patrawadee Yaiwong, Siriporn Anuthum, Padchanee Sangthong, Jaroon Jakmunee, Suwussa Bamrungsap, Kontad Ounnunkad

**Affiliations:** ^1^ Department of Chemistry and Center of Excellence for Innovation in Chemistry, Faculty of Science, Chiang Mai University, Chiang Mai, Thailand; ^2^ The Graduate School, Chiang Mai University, Chiang Mai, Thailand; ^3^ National Nanotechnology Center (NANOTEC), National Science and Technology Development Agency (NSTDA), Pathum Thani, Thailand

**Keywords:** alpha-fetoprotein, detection, aptasensor, screen-printed electrode (SPE), electrochemical biosensor, gold nanoparticles (AuNPs)

## Abstract

The quantification of alpha-fetoprotein (AFP) as a potential liver cancer biomarker which is generally found in ultratrace level is of significance in biomedical diagnostics. Therefore, it is challenging to find a strategy to fabricate a highly sensitive electrochemical device towards AFP detection through electrode modification for signal generation and amplification. This work shows the construction of a simple, reliable, highly sensitive, and label-free aptasensor based on polyethyleneimine-coated gold nanoparticles (PEI-AuNPs). A disposable ItalSens screen-printed electrode (SPE) is employed for fabricating the sensor by successive modifying with PEI-AuNPs, aptamer, bovine serum albumin (BSA), and toluidine blue (TB), respectively. The AFP assay is easily performed when the electrode is inserted into a small Sensit/Smart potentiostat connected to a smartphone. The readout signal of the aptasensor derives from the electrochemical response of TB intercalating into the aptamer-modified electrode after binding with the target. The decrease in current response of the proposed sensor is proportional to the AFP concentration due to the restriction of the electron transfer pathway of TB by a number of insulating AFP/aptamer complexes on the electrode surface. PEI-AuNPs improve SPE’s reactivity and provide a large surface area for aptamer immobilization whereas aptamer provides selectivity to the target AFP. Consequently, this electrochemical biosensor is highly sensitive and selective for AFP analysis. The developed assay reveals a linear range of detection from 10 to 50000 pg mL^−1^ with *R* ^2^ = 0.9977 and provided a limit of detection (LOD) of 9.5 pg mL^−1^ in human serum. With its simplicity and robustness, it is anticipated that this electrochemical-based aptasensor will be a benefit for the clinical diagnosis of liver cancer and further developed for other biomarkers analysis.

## 1 Introduction

Hepatocellular carcinoma (HCC) is one of the most invasive cancers in human beings that occurs very often in patients with chronic liver diseases and cirrhosis. Most common cases of HCC are related to chronic hepatitis B and C virus infections (Marina et al., 2020; Zhang J et al., 2020). Therefore, early HCC diagnosis is essential to the prognosis and survival of patients that can be investigated in laboratory by imaging techniques. However, the imaging technique presents poor diagnostic sensitivity and specificity (Zhang Z M et al., 2020). Consequently, serum biomarker is the most frequently used to predicate HCC. For instance, alpha-fetoprotein (AFP) in human serum is generally regarded as an important biomarker of HCC for targeting liver cancer analysis (Lou et al., 2017; Lee et al., 2019; Li et al., 2021). AFP, a glycoprotein, is usually produced by the fetal liver and digestive tract of humans (Li et al., 2021). In healthy human serum, the AFP level is barely detectable with a concentration lower than 25.0 ng mL^−1^. In approximately 75% HCC patients, the AFP in serum increases apparently to 500 ng mL^−1^ (Li et al., 2018; Li et al., 2021). The existing traditional methods are applied for the diagnosis of liver cancer, including enzyme-linked immunosorbent assay (ELISA) (Preechakasedkit et al., 2018), and chemiluminescence (CL) (Wang et al., 2022), fluorescence (Zhou et al., 2019; Li et al., 2021), colorimetric (Su et al., 2021), and surface-enhanced Raman scattering (Wang et al., 2020) immunoassays. Although these methods offer good sensitivity and selectivity, they perform with utilizing abundant volumes of samples, complex operation, time-consuming, poor recovery, low stability, and high cost (Zhang et al., 2019; Li et al., 2020). Since the AFP is found at trace level, it is undetectable with the common techniques. Thus, the development of high sensitivity and selectivity, rapid, accurate, and low-cost methods for detecting AFP is of the great interest for early cancer diagnosis and monitoring of liver cancer after long-term treatment (Li et al., 2018; Li et al., 2020; Zheng et al., 2020; Li et al., 2021).

In recent years, AFP-specific aptamer has been introduced into the fabrication of biosensors for AFP analysis (Yuan et al., 2021; Chanarsa et al., 2022). Aptamers are short nucleic acid that are single-stranded deoxyribonucleic acid (ssDNA) or ribonucleic acid (RNA) that can specifically bind to their targets (Lerdsri et al., 2020; Li et al., 2020). The binding affinity between the aptamer and the target protein is often stronger than that of the antibody and antigen (Li et al., 2020). When compared to antibodies, aptamer provides good stability, high binding affinity, ease of labeling, and a wide range of targets (Xie et al., 2012; Li et al., 2020). Moreover, the development of aptamer-based biosensors is a critical point together with the high-efficient detection strategies and good signal amplification to assay the biomarker level sensitively and specifically. Electrochemical aptasensing platforms usually have excellent sensitivity, a strong affinity for the target, rapid response, and low cost (Zhang et al., 2019). In previous studies, several electrochemical aptasensors for AFP were successfully developed (Li et al., 2018; Yang et al., 2018; Zhang et al., 2019; Upan et al., 2021). For example, Li et al. (2018) constructed a label-free electrochemical aptasensor based on thionine/reduced graphene oxide/gold nanoparticles for the detection of AFP. The detection is simply achieved via the electrochemical signal change of thionine with the AFP concentration range from 0.1 to 100.0 μg mL^−1^ with the limit of detection (LOD) of 50 ng mL^−1^. Thus, the aptasensor has great potential for screening patients with liver cancer. In addition, Yang et al. (2018) simply developed the label-free aptasensor using graphene oxide for the AFP detection. The aptasensor revealed the dynamic AFP concentration range of 10–100,000 pg mL^−1^ with a LOD value of 3 pg mL^−1^. This sensor revealed the satisfactory analytical performance, simplicity in use, and fast detection that could be applied for early diagnosis or screening of liver cancer (Yang et al., 2018).

The important point for constructing an electrochemical aptasensor is to utilize a modification strategy offering a large surface area and good biocompatibility to improve the efficiency of aptasensing and enhance the detection performance (Yang et al., 2018; Zhang et al., 2019; Li et al., 2020). Disposable three-electrode screen-printed electrodes (SPEs) have been used in the field of device technology for making the electrochemical aptasensor due to their simplicity, ease of use, cost-effectiveness, and high reproducibility (Upan et al., 2021). Especially, they can be implemented as portable aptasensors when applied with a portable potentiostat (Chanarsa et al., 2022). Gold nanoparticles (AuNPs) have attracted great interest because of their unique physical and chemical properties, which exhibit the potential use for chemical and biological detection due to their nanoscale size, large surface area, and high conductivity (Ding et al., 2019; Khan et al., 2019). In our previous studies, poly (ethyleneimine)-coated-AuNPs (PEI-AuNPs) were utilized as an electrode-surface modifier and an antibody-conjugation support in electrochemical sensors for the detection of lung and breast cancer biomarkers (Kuntamung et al., 2021a; Kuntamung et al., 2021b). The PEI-capped AuNPs were generated via the reduction of AuCl_4_^−^ anions to Au^0^ atoms on protonated amine cations of PEI via an amide linkage, thus offering stability in aqueous solutions (Ortega-Munoz et al., 2016; Hu et al., 2017). Interestingly, PEI coated on AuNPs not only gives strong adsorption and high stability but also helps in AuNPs’ binding efficiency with metal, signaling molecules, antibodies, proteins, and aptamers (Ortega-Munoz et al., 2016; Hu et al., 2017; Kuntamung et al., 2021a; Kuntamung et al., 2021b). For the label-free system, the sensors rely on the redox-active signal in the solution of K_3_[Fe(CN)_6_] and K_4_Fe(CN)_6_ species, which changes upon the capturing of target proteins. Nevertheless, the activity of biomolecules is usually significantly reduced due to the presence of the solution-phase external mediator (Kuntamung et al., 2021a). Therefore, the development of label-free electrochemical aptasensors with the attached redox-active mediators on the electrode surface can improve the stability of their electrochemical responses (Kuntamung et al., 2021b; Yaiwong et al., 2021). Toluidine blue (TB) is a basic thiazine metachromatic dye that has a good affinity for nucleic acid sequences (Sridharan and Shankar, 2012; Ding et al., 2019). Additionally, TB is widely used as a plausible redox probe in electrochemical aptasensor due to its good electron transfer ability and great conductivity (Peng et al., 2015). The possible mechanism of TB and aptamer (short nucleic acid sequences) interaction is proposed that TB containing amino groups can intercalate to nucleic acid molecules of the aptamer through electrostatic interaction (Sridharan and Shankar, 2012; Ding et al., 2019).

In the sensor development, although the electrochemical sensors generally achieve very low LOD and high sensitivity, they must be simple, selective, stable, and cost-effective. In this work, a simple label-free electrochemical aptasensor based on TB/BSA/APt/PEI-AuNPs-modified SPE with high affinity towards AFP assay is successfully developed, which performs on a small Sensit/Smart potentiostat connected to a smartphone, for the first time. A disposable SPE electrode was sequentially deposited with PEI-AuNPs, aptamer, BSA, and TB, respectively. The electrochemical signal was generated from the TB intercalating into an aptamer-bounded electrode platform and amplified its intensity corresponding to the change in the AFP content. After the aptamer recognizes AFP at its specific recognition units, the current response of the proposed sensor is reduced proportionally to the AFP concentration, implying that the AFP/DNA aptamer complexes restrict the electron transfer of TB. The sensor reveals acceptable selectivity, sensitivity, reproducibility, recovery result, and stability. Moreover, the proposed aptasensor strategy can be further applied to the clinical diagnosis of liver cancer. From our findings, by replacing AFP-related DNA aptamer with a variety of other diseases-involving aptamers, this sensor technology development strategy as a model can operate in the detection of their biomarkers as well as in the clinical diagnoses of their corresponding diseases, especially other cancers.

## 2 Experiment

### 2.1 Materials and reagents

Alpha-fetoprotein (AFP, lot: A16042812, 6.9 mg mL^−1^, >98%) was purchased from Fitzgerald Industries International (United States). Gold (III) chloride hydrate (HAuCl_4_⋅H_2_O, 99.995%), phosphate buffer saline tablets (PBS, 0.010 M, pH 7.4), male human serum (AB plasma, United States origin, lot: SLBS6544), *L*-ascorbic acid (AA, lot: STBC8330V, 99%), *D*-(+)-glucose (Glu, lot: SLBR5156V, ≥99.5%), immunoglobulin G (IgG, lot: 082M4772V), interleukin 6 human (IL-6, lot: 0409AFC16), carcinoembryonic antigen (CEA, lot: SLBM6069V), and matrix metalloproteinase-7 human (MMP-7, lot: SLBW7614) were purchased from Sigma-Aldrich (United States). Dopamine hydrochloride (DA, lot: BCBS3110, 98%) was bought from Sigma-Aldrich (Germany). Recombinant human GM2A protein (GM2AP, lot: E322118929) was achieved from Abcam (United States). Recombinant human HER2/ErbB2 protein (HER2, lot: LC11MC0201) was bought from Sino Biological Inc. Bovine serum albumin (BSA, lot: D00155527, >98%) was purchased from Calbiochem (Germany). Sodium dihydrogen phosphate dihydrate (NaH_2_PO_4_⋅2H_2_O, 99.0–101.0%) was ordered from Scientific (United States). Disodium hydrogen phosphate dihydrate (Na_2_HPO_4_⋅2H_2_O, 99.5%) was acquired from QRëC (New Zealand). Polyethylenimine (PEI, branched, *M*_w_ = 800) and 75-mer AFP-specific aptamer oligonucleotide (lot: SG00398903, *M*_w_ = 23016) purified with HPLC and the following sequence,5′-GTGACGCTCCTAACGCTGACTCAGGTGCAGTTCTCGACTCGGTCTTGATGTGGGTCCTGTCCGTCCGAACCAATC-3′), were obtained from Sigma-Aldrich (Singapore). Toluidine blue (TB, 98.0%) and Potassium ferricyanide (K_3_[Fe(CN)_6_], 99%) were purchased from Merck (Germany). All aqueous solutions were prepared using deionized (DI) water. The aptamer solution and working solutions (*e.g.*, BSA, AFP, and interference substances) were diluted in DI water and phosphate buffer solution (PB, 0.010 M, pH 7.0), respectively. Then, the prepared solutions were stored at 4°C if not in use.

### 2.2 Instrumentation and measurements

For material characterization, preliminary device construction, and optimization of device fabrication parameters, the electrochemical measurements using a three-electrode 5-mL electrochemical cell setup were conducted on an EmStat3 and PalmSens4 potentiostats (PalmSens, Netherlands) with PSTrace 5.8 software. The three-electrode setup is composed of a home-made screen-printed carbon electrode (SPCE, 3.0 mm in diameter) (Kuntamung et al., 2021a; Phetsang et al., 2021; Yaiwong et al., 2021), a platinum wire (Nilaco Co. Ltd., Japan), and a silver/silver chloride electrode (Ag/AgCl, 3M NaCl) (BASi, United States), which acted as working, counter, and reference electrodes, respectively. For the construction of the optimized aptasensor and the investigation of its analytical performance, a small potentiostat (Sensit/Smart, PalmSens, Netherlands), affixed with a smartphone (Xiaomi Redmi 9), and controlled by the Android application (PStouch), was employed. An ItalSens screen-printed electrode (SPE, PalmSens, Netherlands) used in this study is compatible with the portable potentiostat. The ItalSens SPE also consists of a graphite working electrode (3 mm in diameter), a silver/silver chloride pseudo-reference electrode, and a graphite counter electrode. Cyclic voltammetry (CV) and electrochemical impedance spectroscopy (EIS) were used for the electrochemical characterization in 0.010 M PBS (pH 7.4) containing 5.0 mM [Fe(CN)_6_]^3−^. CV measurements were recorded with a potential scan range from −0.4 to +0.8 V at a scan rate of 50 mV s^−1^. All EIS experiments were carried out at a frequency in the range of 10^2^–10^5^ Hz with an alternating current amplitude of 10 mV. For the optimization of the aptasensor fabrication, differential pulse voltammetry (DPV) measurements in contact with 0.010 M PBS (pH 7.4) were employed by setting the potential range from −1.0 to 0.20 V. A potential step of 0.010 V, a modulation amplitude of 0.20 V, a modulation time of 20 m, and a scan rate of 50 mV s^−1^ were applied. Furthermore, morphology and size of the synthesized PEI-AuNPs were studied using a scanning electron microscope (SEM-EDS; JEOL JSM-IT300LV, Japan) and a transmission electron microscope (TEM, JEM-2010, Japan). A UV-Visible (UV-vis) spectrum was recorded by an Evolution 201 Spectrophotometer (Thermo Scientific, United States) over the wavelength range of 200–750 nm. Centrifugation was executed using a Digicen 21 Centrifuge (Orto Alresa, Spain).

### 2.3 Fabrication of the aptasensor and its AFP detection

**SCHEME 1:**
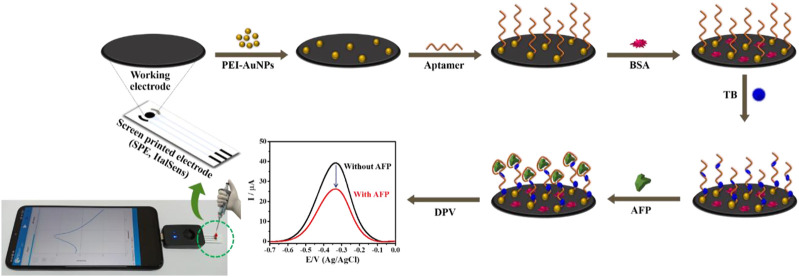
Illustration of the fabrication steps of proposed label-free electrochemical aptasensor for AFP detection.

PEI-AuNPs were synthesized under mild reaction condition in our laboratory, according to the previous reports (Putnin et al., 2019; Kuntamung et al., 2021a; Kuntamung et al., 2021b). PEI was employed as the reducing and stabilizing agent. A PEI-AuNPs solution was preserved at 4°C before further use. Before the aptasensor construction, SPCE and the working electrode of SPE were treated with O_2_ plasma at 400 mbar for 1 min by using a plasma cleaner (PDC-32G, Harrick Plasma, United States). The construction process of a label-free electrochemical aptasensor is displayed in Scheme 1. Briefly, the treated working electrodes were modified by 2.5 μL of PEI-AuNPs solution and allowed to dry at an ambient temperature. Subsequently, 2.5 μL of 10 mM aptamer (Apt) was immobilized onto the PEI-AuNPs modified electrode for 30 min in a humidity chamber at room temperature, followed by washing with DI water 3 times to remove unbound aptamer. To block non-specific adsorption of other proteins or non-target molecules and the remaining active sites of the Apt/PEI-AuNPs modified SPE, the electrode surface was incubated with 5.0 μL of 0.5%w/v BSA solution for 30 min in the chamber at a room temperature. The obtained BSA/Apt/PEI-AuNPs modified SPE was rinsed with 0.010 M PB (pH 7.0) several times. Next, 5.0 μL of 15 mM TB was adsorbed onto the BSA/Apt/PEI-AuNPs modified SPE with the incubation time of 20 min at a room temperature, and then the resultant electrode was washed with 0.010 M PB (pH 7.0) for 3 times. Finally, the aptasensor was kept in a refrigerator at 4°C when not in use. To detect AFP, the TB/BSA/Apt/PEI-AuNPs modified SPEs were incubated with 2.5 μL of the AFP solutions at different concentrations for 30 min at room temperature, followed by washing with 0.010 M PB (pH 7.0) 3 times to get rid of the unbound AFP antigen. The responses of the AFP aptasensors after incubation with the AFP solutions (0.010–50 ng mL^−1^) were measured using DPV in contact with 0.010 M PB (pH 7.0) to construct a calibration curve. To mimic clinical sample analysis, AFP was spiked into 50-fold diluted human serum at certain concentrations.

## 3 Results and discussion

### 3.1 Morphological characterization of the modified electrode

**FIGURE 1:**
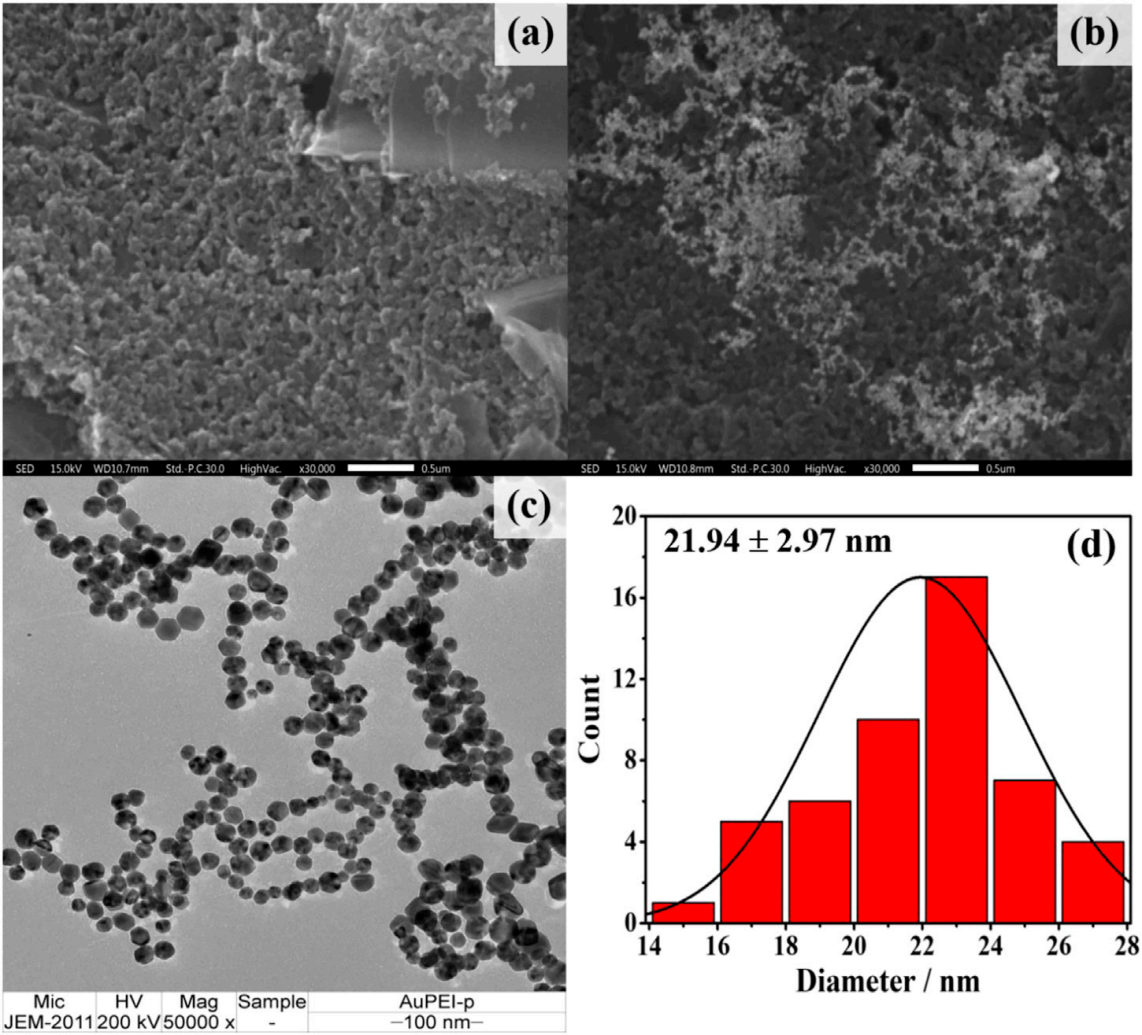
SEM images of **(A)** naked and **(B)** PEI-AuNPs modified SPCEs **(C)** TEM image of the synthesized PEI-AuNPs and **(D)** corresponding histogram of size distribution profile (21.94 ± 2.97 nm).

Before electrochemical aptasensor fabrication, PEI-AuNPs were modified onto the electrode surface to offer improved electrochemical reactivity and receptor immobilization capacity (Putnin et al., 2019). The solution of PEI-AuNPs has high stability under a storage condition at 4°C (Thiramanas and Laocharoensuk, 2016; Putnin et al., 2019). The morphology of PEI-AuNPs-modified working electrode was investigated by SEM, while TEM and UV-Vis spectroscopy techniques were used to analyze the size and light absorption of the PEI-AuNPs, respectively. SEM images of both unmodified and modified working electrodes with PEI-AuNPs under the same magnification were displayed in Figures 1A, B, respectively. The carbon particles of an unmodified working electrode are inhomogeneous and show a porous structure covered by small and large carbons with irregular particle shapes (Figure 1A) (Dong et al., 2007). After PEI-AuNPs modification, SEM confirmed that the presence of PEI-AuNPs on top of the working electrode. Then, the size and shape of synthesized PEI-AuNPs used in this study were further confirmed by TEM image as shown in Figures 1C, D, respectively. The TEM image (Figure 1C) reveals good dispersion and uniform particles of quasi-spherical shape, and the average diameter of PEI-capped AuNPs distributed is about 21.94 ± 2.97 nm (see the average size histogram in Figure 1D) (Bali et al., 2021). The average size of PEI-AuNPs depends on the quantity of reducing agent and reaction time which is likely related to the reduction process of Au(III) ions (HAuCl_4_) with the amine groups of PEI (Kim et al., 2018). It has been observed that the maximum peak obtained in the UV-vis absorption spectrum for PEI-AuNPs is at 524 nm, as depicted in Supplementary Figure S1 (supporting information). This result indicates that the absorption peak is related to the mean diameter value, size, and shape of PEI-AuNPs (Gurunathan et al., 2014). The distribution of PEI-AuNPs observed by the SEM and TEM techniques would lead to a larger specific surface area of the modified electrode, thereby improving the electrochemical properties, the amount of adsorbed active biomolecules, and the sensitivity of detection.

### 3.2 Label-free electrochemical aptasensor characterization

**FIGURE 2:**
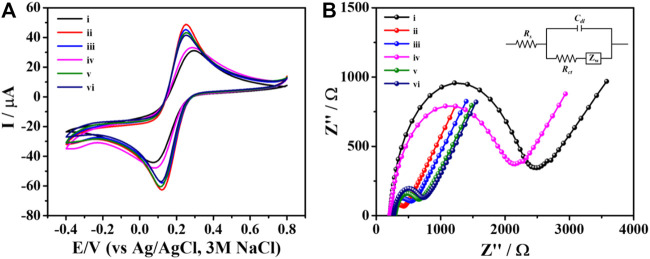
**(A)** CVs and **(B)** EIS spectra of the different modified electrodes for each modification step: (i) naked SPCE, and (ii) PEI-AuNPs-, (iii) Apt/PEI-AuNPs-, (iv) BSA/Apt/PEI-AuNPs-, (v) TB/BSA/Apt/PEI-AuNPs-, and (vi) AFP/TB/BSA/Apt/PEI-AuNPs-modified SPCEs, recorded in contact with PBS (0.010 M, pH 7.4) containing 5.0 mM [Fe(CN)_6_]^3−^.

**FIGURE 3:**
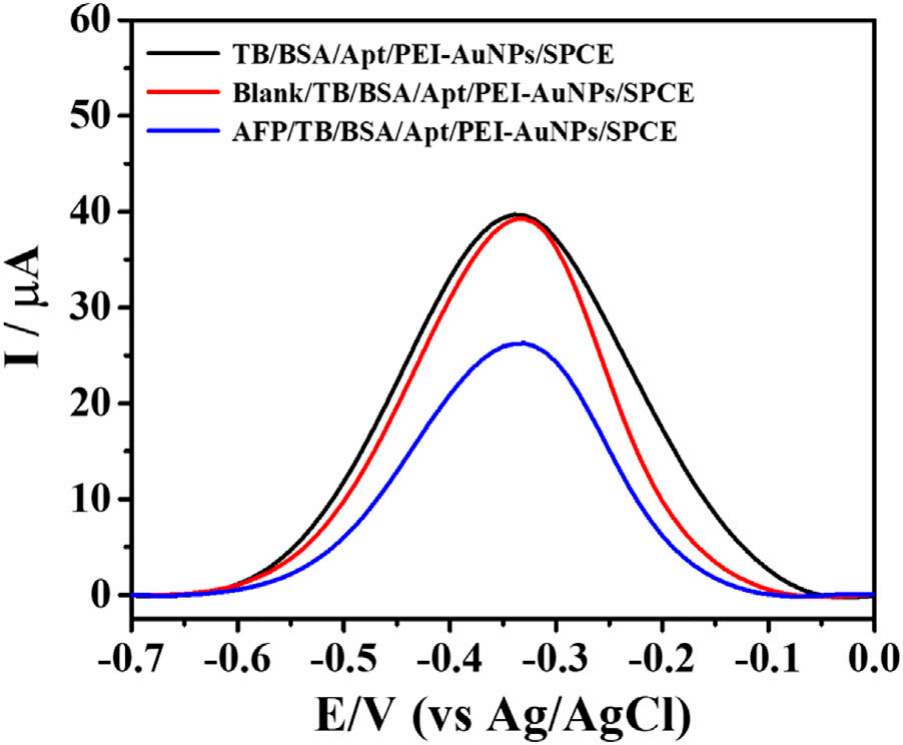
DPV responses of the sensing TB/Apt/BSA/PEI-AuNPs-modified electrodes with and without capturing AFP, measured in PB buffer solution (0.010 M, pH 7.0).

The CV and EIS methods were applied to monitor behavior of the label-free electrochemical aptasensor in each step of the fabrication. The CV responses of the naked SPCE (curve i), PEI-AuNPs/SPCE (curve ii), Apt/PEI-AuNPs/SPCE (curve iii), BSA/Apt/PEI-AuNPs/SPCE (curve iv), TB/BSA/Apt/PEI-AuNPs/SPCE (curve v), and AFP/TB/BSA/Apt/PEI-AuNPs/SPCE (curve vi) were studied using 0.01 M PBS (pH 7.4) solution containing 5.0 mM [Fe(CN)_6_]^3−^ redox probe. The applied potential was ranging from −0.40 to 0.80 V with a scan rate of 50 mV s^−1^. The quantitative data were collected and observed, including the peak-to-peak separation (∆*E*_p_ = *E*_pa_ - *E*_pc_), oxidation peak current (*I*_pa_), and reduction peak current (*I*_pc_); these were performed by changing the loading component on the SPCE’s surface above, as can be seen in Figure 2A. The PEI-AuNPs/SPCE (curve ii) exhibits excellent oxidation peak current at 63.46 μA and shows a lower ∆*E*_p_ (0.130 V) than naked SPCE (curve i, *I*_pa_ = 39.04 μA, and ∆*E*_p_ = 0.210 V) because of its high conductivity as well as great electron transfer ability (Kuntamung et al., 2021a; Kuntamung et al., 2021b). The AFP-specific ssDNA aptamer was selected as a bioreceptor with a high affinity towards the target antigen (AFP) with the *K*_d_ value of 2.37 nM (Huang et al., 2012; Dong et al., 2015). After immobilization of aptamer on the surface of PEI-AuNPs/SPCE (curve iii), the oxidation peak current was slightly decreased (58.36 μA), suggesting that the immobilized aptamer reduced electron transfer at the electrode/electrolyte interface (Upan et al., 2021). Subsequently, the oxidation peak response was further decreased to 42.01 μA and the ∆*E*_p_ value increased to 0.190 V after attaching BSA (curve iv) because the non-specific aptamer binding sites were blocked. As expected, the *I*_pa_ value increased (57.00 μA) and the ∆*E*_p_ value decreased (0.140 V) after incubation of TB (curve v). Herein, TB could be served as an electrochemical indicator for the detection and the increase in current at the presence of TB on the modified electrode could also enhanced the electrochemical sensitivity (Xie et al., 2012; Li et al., 2018; Ding et al., 2019). When AFP protein was incubated on the surface of TB/BSA/Apt/PEI-AuNPs/SPCE, the redox peak current was slightly decreased (curve vi, 54.10 μA) suggesting the complex formation between AFP and aptamer. The resultant complexes hindered electron transfer and mass-transfer of the modified electrode. These results confirmed that the AFP molecules were bound with aptamers, thereby the aptasensor has been successfully constructed. In addition, the Nyquist plot was done to investigate the interface properties of the fabricated electrodes. The result was correlating with the CV measurement as seen in Figure 2B which further confirm the sensor construction process. It is well known that the frequency response in Nyquist plots exhibits a semicircle part at high frequencies which refers to the electron-transfer resistance (*R*_ct_) at the electrode interface. While the linear part at lower frequencies demonstrates the diffusional-limited electron-transfer process (Haji-Hashemi et al., 2018). The *R*_ct_ of the unmodified SPCE exhibited the largest semicircle (curve i, 2,155 Ω) suggesting the regression of the electron transfer process because of the low conductivity of the naked SPCE (Yaiwong et al., 2021). On the other hand, after the naked SPCE was modified with PEI-AuNPs, curve ii exhibited the lowest semicircle domain and showed an *R*_ct_ value of 203 Ω demonstrating that AuNPs promoted excellent electron transfer and great conductivity on the modified electrode surface (Sangili et al., 2020). By the aptamer immobilization, the electron transfer was hindered by the aptamer, and the *R*_ct_ value increased up to 305 Ω (curve iii). Furthermore, the semicircle and the *R*_ct_ value were immediately increased (1946 Ω, curve iv) after the blocking step. The increment of the semicircle and *R*_ct_ value can be explained by the blocking of the modified electrode surface by the BSA resulting in less accessible of the [Fe(CN)_6_]^3−^ redox coupling (Jampasa et al., 2019). When TB was adsorbed onto the modified electrode, the semicircle diameter decreased drastically (*R*_ct_ = 413 Ω, curve v), which was attributed to the raise of electric conductivity of TB that can give a fast electron transfer at the interface/electrolyte (Wang et al., 2017). Afterward, the specific interaction between the aptamer and AFP occurred, and the modified electrode showed an increasing resistance (*R*_ct_ = 509 Ω, curve vi). This demonstrated that the formation of the insulating layer interrupted the diffusion of electron/ferricyanide toward the electrode surface. The obtained EIS results were in agreement with those from the CV, thus the label-free electrochemical aptasensor was successfully developed. The detection of AFP by the proposed aptasensor was then validated using DPV measurement in 0.010 M PB buffer solution (pH 7.0) as demonstrated in Figure 3. After a TB/Apt/BSA/PEI-AuNPs-modified electrode was incubated with a blank solution, the current response (*ca*. 39 μA) was not significantly changed. Furthermore, the response was dramatically decreased after capturing 50 ng mL^−1^ AFP. It is plausible that the layer of AFP/aptamer complexes on the surface increases the distance of the electrode contact resulting in a reduced electrochemical signal (*ca*. 26 μA) (Shao et al., 2018; Upan et al., 2021).

### 3.3 Optimization conditions for electrochemical aptasensor

The optimization of the PB’s pH in the range of 5.0–8.0 was studied as the first step to improve the detection sensitivity as seen in Supplementary Figure S2 (supporting information). It is noted that the suitable pH value would offer good reactivity and stability of the aptamer. This gives a stable analytical current. The aptasensors, prepared with 2.5 μL of PEI-AuNPs, 2.5 μL of 10 μM aptamer, 2.5 μL of 0.5% w/v BSA, and 2.5 μL of 15 mM TB under the incubation times of 30 min, were used for this study. After binding with 50 ng mL^−1^ AFP, the result showed that the maximum current change (∆I, *ca*.12.7 μA) could be obtained with a pH 7.0, thus suggesting the best bioreactivity of the aptamer (Yang et al., 2018). Moreover, the optimization of each fabrication step is required including the aptamer concentration and immobilization time, TB concentration and adsorption time, as well as the AFP capture time. All optimal parameters could maximize the current change which reflects the sensitivity of the aptasensor for the target determination.

**FIGURE 4:**
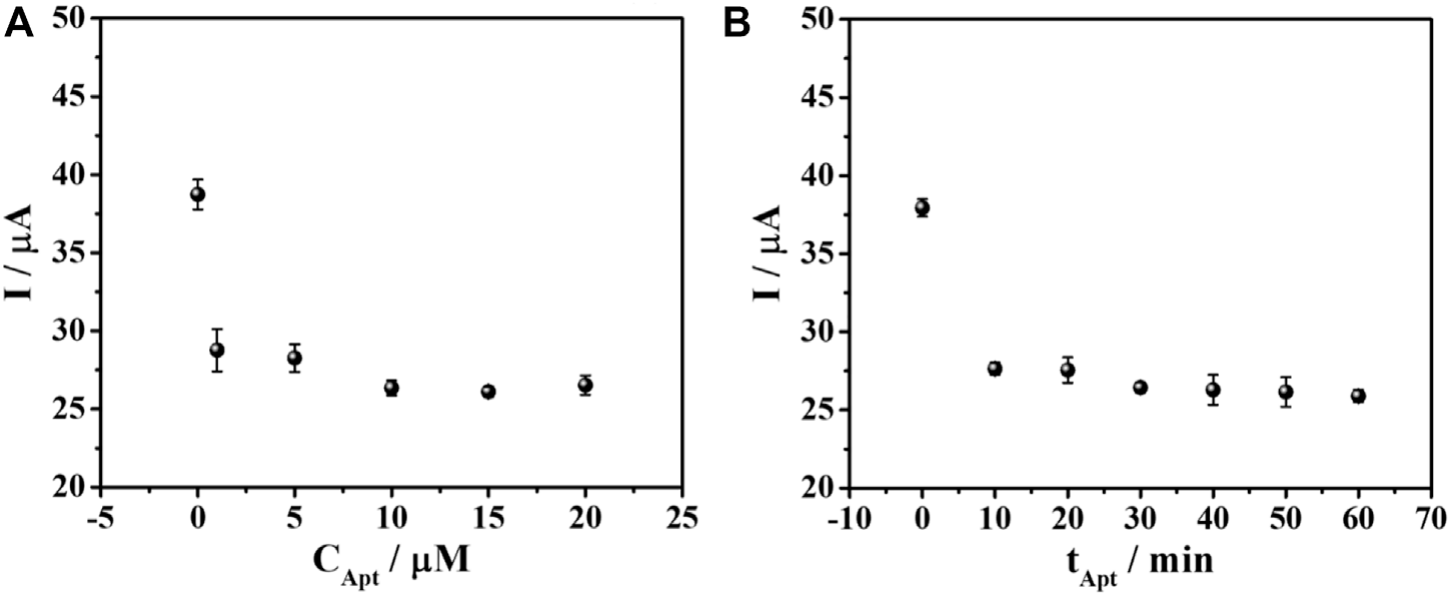
Effect of **(A)** aptamer concentration and **(B)** immobilization time on the DPV current responses of TB for the aptasensor after binding with AFP measured in PB (0.010 M, pH 7.0).

The optimal concentration for aptamer loading was investigated based on the current response of the quantitative detection of 50 ng mL^-1^ AFP. The adsorption of aptamer on AuNPs is simply obtained by electrostatic interaction (Hu et al., 2018; Mirau et al., 2018; Rashid et al., 2020). The aptasensors were fabricated with a similar condition to the previous experiment except various concentrations of aptamer ranging from 1 to 20 µM were used. Then, the AFP detection was observed by DPV in 0.010 M PB buffer solution (pH 7.0) and the result was demonstrated in Figure 4A. It was shown that at 10 µM aptamer, a decrease in current starts to be constant. This indicates the saturation of aptamer on the electrode surface. Therefore, 10 µM of aptamer was chosen for immobilization on the PEI-AuNPs-modified SPCE. The immobilization time of aptamer was also studied in the range of 10–60 min, as illustrated in Figure 4B. The current reduction reached a minimum at 30 min of incubation and kept constant after this time point. This suggested the maximum loading of the aptamer that could provide the best sensitivity due to the complete aptamer loading.

**FIGURE 5:**
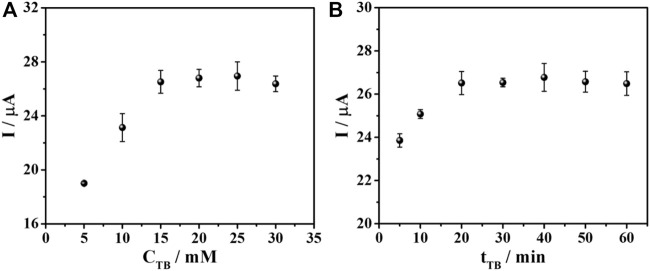
Effect of **(A)** TB concentration and **(B)** TB adsorption time on DPV current responses for the aptasensor after binding with AFP in PB (0.010 M, pH 7.0).

For the sensor fabrication, the PEI-AuNPs-modified SPCE is incubated with the DNA aptamer and then BSA, to block the direct adsorption of the redox indicator (TB). Consequently, TB is expected to infiltrate only the DNA aptamer structure (Chanarsa et al., 2022). According to the generation of the current response and the signal amplification, the adsorption amount of TB affects the measurement sensitivity and signal stability. In addition, TB redox response can quantitate target AFP protein concentration. The protein is captured by aptamer, which builds up the layer of an insulator AFP/aptamer complexes, restricting the electron transfer at the electrode. As a result, the current is decreased, corresponding to the surface-bound complex content. The sensors prepared with the optimized condition were employed for the TB adsorption study using various concentrations of TB in the range of 5–30 mM as depicted in Figure 5A. It is found that after 50 ng mL^-1^ AFP is captured on the electrode surface, the current response is a function of the TB concentration. The current response increased and then reached a plateau at 15 mM (*ca*. 26 μA), indicating no further TB uptake. This concentration gave the maximum loading of TB as well as the maximum current. The TB concentration of 15 mM was therefore, chosen for further optimization. Moreover, the adsorption time of TB from 5 to 60 min was also investigated. The current response varied regarding the incubation time, as seen in Figure 5B. The periods from 20 to 60 min yielded an unchanged signal (*ca*. 26 μA) caused by the complete TB adsorption. The highest and most stable response of the sensor is obtained with the incubation time of 20 min, which will be applied for the next study.

**FIGURE 6:**
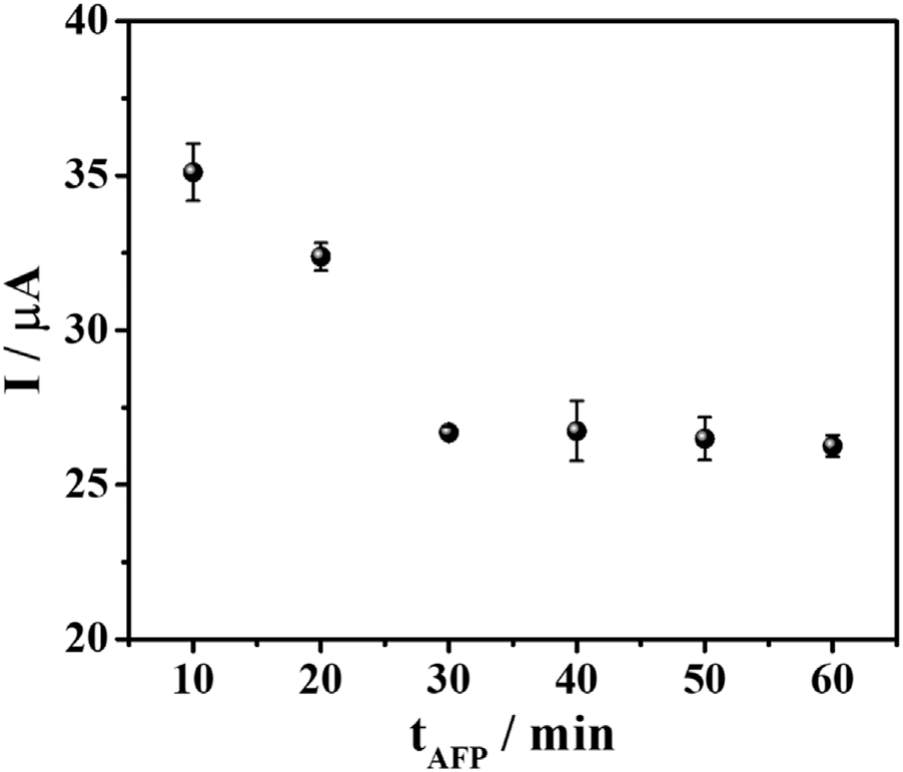
A plot between AFP incubation time and DPV current responses of TB after binding with 50 ng mL^-1^ AFP in PB (0.010 M, pH 7.0).

Additionally, the AFP binding time was optimized to achieve the best analytical performance of the sensor. Different incubation times (10–60 min) were applied to allow the binding between the aptamer and the target AFP. The result in Figure 6 revealed that the current response reduced by time and reached the minimum (*ca*. 26 μA) until constant at 30 min. This resulted from the complete AFP/aptamer complex formation on the aptamer-saturated electrode which suggested that the sensor fabricated from this condition would offer the best detection performance. Consequently, the aptasensor will be further fabricated and applied for AFP determination under these all-optimized conditions.

### 3.4 Analytical performance of aptasensor

**FIGURE 7:**
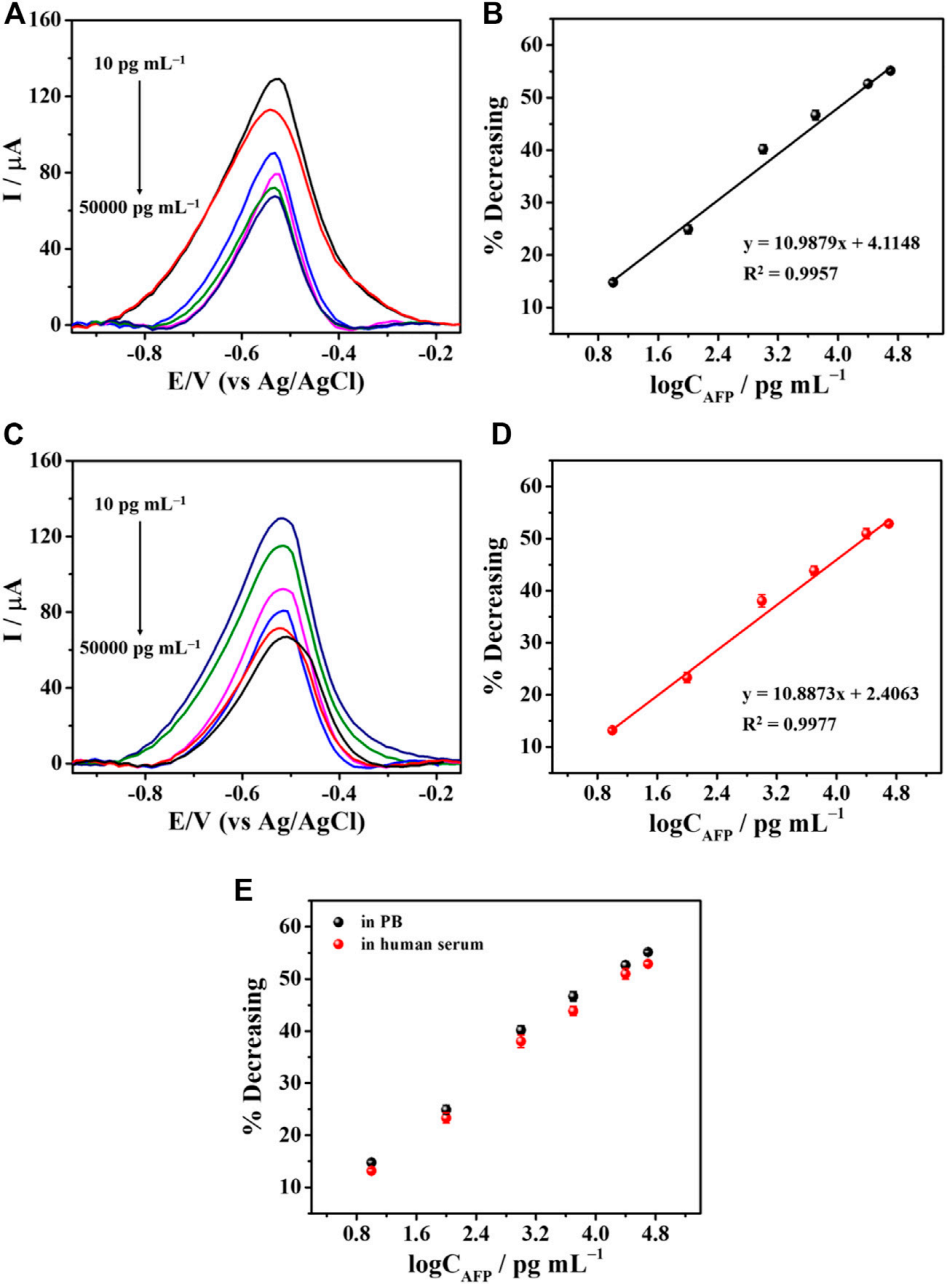
The detection of AFP in PB and serum. **(A)** DPV responses to difference concentrations of AFP in PB and **(B)** their related % current decreasing. **(C)** DPV responses to various AFP concentrations in serum and **(D)** their corresponding % current reduction. **(E)** A comparison of calibration curves derived from PB and human serum.

Under optimal conditions, the fabricated aptasensor based on SPE was used to determine AFP via the observation of the TB oxidation peak using DPV technique. The data achieved from various AFP concentrations were recorded by inserting the sensor into the smartphone and running via a PStouch application. The ability to detect AFP by our aptasensors is tested in two different matrices: AFP-spiked PB and 50-fold diluted human serum solutions, respectively (Figure 7). It is found that with increasing the AFP concentration, a decrease in TB signal was observed proportionally as described in Figure 7A (PB) and Figure 7C (human serum). The concentrations ranged from 10 to 50,000 pg mL^−1^ AFP were employed, and the % decreasing of the current peak *versus* the logarithm of the concentration showed a good linear relationship for both cases (Figures 7B, D). According to Figures 7A, B calibration curve with a linear regression value (*R* ^2^) of 0.9957 over the concentration range (10–50,000 pg mL^–1^), and a LOD value (3*SD*_blank_/slope) of 8.8 pg mL^-1^ was observed for the AFP detection in PB. Whilst the AFP analysis in serum in Figure 7D, an *R* ^2^ value and a LOD value were 0.9977 and 9.5 pg mL^−1^, respectively. Interestingly, both calibration curves superimpose, as shown in Figure 7E, indicating the high selectivity of our sensor over many kinds of other real interfering redox and non-redox biochemical species in human serum. Moreover, this figure can imply that the sensor would perform the AFP detection with high accuracy.

Supplementary Table S1 shows the comparison of the performance of our device with other previous reports. Although our sensor has less complexity, its performance is comparable and acceptable, offering the ability to detect AFP. In the optical strategies, Entries 1–3 are higher LOD values than that of the developed AFP aptasensor while the dynamic range of Entry 3 is wider. The sensors could not detect AFP at the ultratrace levels. These aptasensors have several drawbacks, such as time-consuming, complicated preparation, high-volume usage, limited application, and high cost. In addition, their signals might be interfered with matrix colors, suggesting poor detection efficiency. As compared with another three electrochemical aptasensors (Entries 5, 6, and 9), the LOD value of the proposed aptasensor is comparable. However, the dynamic linear range of Entry 9 is shorter, thus resulting in limited applicable use. Furthermore, Entry 6 is operated with multiple-step strategies causing difficulty in AFP detection. Entries 4, 7, and 8 reveal higher LODs, and only Entries 7–8 are demonstrated with larger dynamic ranges, but their ranges start at high concentrations. These two sensors could not detect the protein at the ultratrace levels. For these reasons, our aptasensor with simple preparation, easy operation, and cost-effectiveness would be an alternative tool to detect AFP for early clinical diagnosis of liver cancer.

### 3.5 Reproducibility, specificity, and stability of aptasensor

**FIGURE 8:**
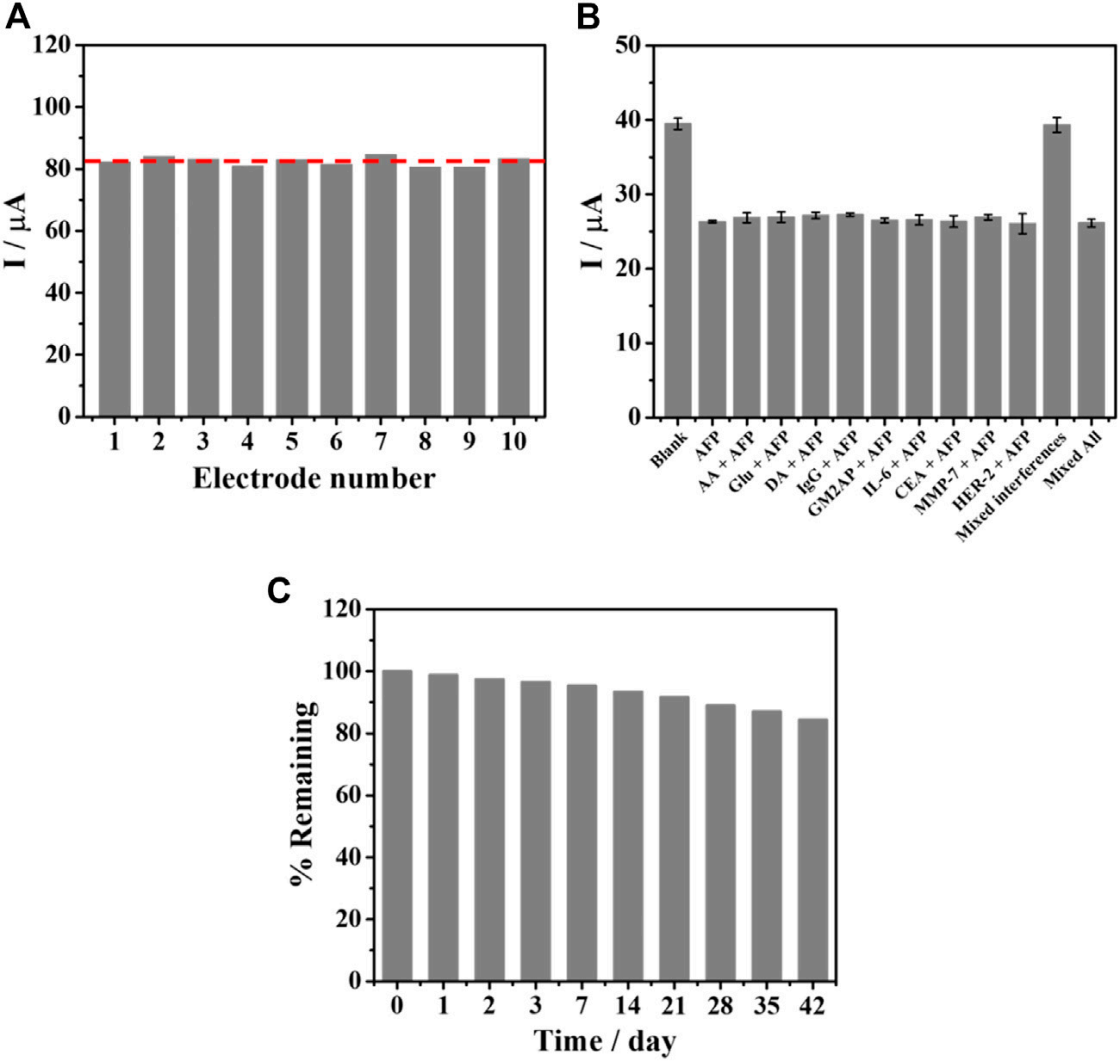
**(A)** Reproducibility of the TB/BSA/Apt/PEI-AuNPs-based aptasensor, after binding with 5.0 ng mL^−1^ AFP, **(B)** interference study for the detection of 5.0 ng mL^−1^ AFP at the presence of interferences including AA, Glu, DA, IgG, GM2AP, IL-6, CEA, MMP-7, HER-2, mixture of interferences, and mixture of interferences without AFP, and **(C)** stability test for the aptasensors over 42 days.

To evaluate the applicability, the aptasensor’s reproducibility, specificity, and stability were studied as described in Figure 8. Ten individual aptasensors based on SPE were tested by capturing 5.0 ng mL^−1^ AFP as shown in Figure 8A. After AFP was bound to the TB/BSA/Apt/PEI-AuNPs-modified SPE, the result showed no significant difference in sensing responses (*ca*. 82 μA) with the RSD value of 1.86%. In addition, as shown in Supplementary Figure S3 (supporting information), RSD values for the detection of 0.1 ng mL^−1^ and 50 ng mL^−1^ AFP are 1.60% and 2.22%, respectively, indicating good reproducibility. Since human blood contains many types of proteins, the specificity of the SPCE-based aptasensor was also examined toward the detection of 5.0 ng mL^−1^ AFP with and without 100-fold higher concentration of interfering substances, as seen in Figure 8B. The capturing studies of blank solution and interference mixture solutions with and without 5.0 ng mL^−1^ AFP were also carried out. The redox and non-redox interferences used in this study consisted of AA, Glu, DA, IgG, GM2AP, IL-6, CEA, MMP-7, HER-2, and their mixture. With the addition of AFP into the solutions, their responses were reduced and insignificantly changed (*ca*. 26 μA) as compared to those of the blank solution, and the interference mixture with the absence of AFP (*ca*. 39 μA). This implies that only AFP/aptamer complexes formed without nonspecific adsorption of all interferences and hindered the electron transfer process of TB resulting in lowering the current response. This result indicated that our sensor provided high detection selectivity. Figure 8C shows the stability of the aptasensors under 4°C storage over 6 weeks by evaluating current signal when the AFP was bound onto the electrode surface. The decline in the current response of the aptasensor during the AFP detection was observed as a function of the time (day). After 42 days storage, the response remains 84% of the initial value, suggesting acceptable stability.

### 3.6 Application in human serum analysis

To evaluate the practical application of the proposed aptasensor, it was applied to the detection of AFP in a 50-fold diluted human serum sample. The AFP-spiked standard solutions at different concentrations (2.5, 5.0, 10.0, and 15.0 ng mL^−1^) were employed. The acceptable recoveries and RSDs of four spiked AFP concentrations were obtained with 99.81%–105.95%, and 0.65%–2.26%, respectively, as demonstrated in Table 1. Therefore, it has the feasibility for application in liver cancer diagnostics.

**TABLE 1:** The application of the proposed PEI-AuNPs aptasensor for determination of human serum samples spiked with difference concentrations of AFP.

Samples	Added AFP (ng mL^-1^)	Detected AFP (ng mL^-1^)	RSD (%)	Recovery (%)
diluted serum 1	2.5	2.55 ± 0.02	0.83	102.03
diluted serum 2	5.0	4.98 ± 0.11	2.26	99.81
diluted serum 3	10.0	10.10 ± 0.07	0.65	101.02
diluted serum 4	25.0	26.48 ± 0.26	0.96	105.95

## 4 Conclusion

In summary, a new portable TB/aptamer complex-PEI-AuNPs-based electrochemical aptasensor attached to an android smartphone is developed for the sensitive and selective detection of AFP in the label-free amplification format by using an intercalating TB response as an analytical signal. The blockage of electron transfer of TB due to capturing the insulating and bulky target AFP molecules by aptamer on the electrode is proportional to an amount of surface-confined AFP content. The demonstrated aptasensor has high sensitivity and can detect AFP at the clinically relevant concentration in human serum. Moreover, the aptasensor shows a LOD value of 9.5 pg mL^–1^AFP, with a wide dynamic range of 10–50,000 pg mL^–1^ AFP in diluted human serum. The developed sensor offers excellent selectivity, stability, and reproducibility. Our new aptasensor has the potential as an alternative tool for liver cancer diagnosis as well as a model for further development of other aptasensors in the detection of other cancer biomarkers.

## Data Availability

The original contributions presented in the study are included in the article/Supplementary Material, further inquiries can be directed to the corresponding author.
