# Supplementary material for: A new portable toluidine blue/aptamer complex-on-polyethyleneimine-coated gold nanoparticles-based sensor for label-free electrochemical detection of alpha-fetoprotein
Source: Front Bioeng Biotechnol. 2023 May 22;11:1182880. doi: 10.3389/fbioe.2023.1182880 (PMC10239980; doi:10.3389/fbioe.2023.1182880)
Supplement: Supplementary file 1 [file DataSheet1.docx]

Supplementary Material

A New Portable Toluidine Blue/Aptamer Complex-on-Polyethyleneimine-Coated Gold Nanoparticles-Based Sensor for Label-Free Electrochemical Detection of Alpha-fetoprotein

Patrawadee Yaiwong^1,2^, Siriporn Anuthum^1,2^, Padchanee Sangthong^1^, Jaroon Jakmunee^1^, Suwussa Bamrungsap^3^, Kontad Ounnunkad^1*^

* Correspondence: Corresponding Author: suriyacmu@yahoo.com, kontad.ounnunkad@cmu.ac.th


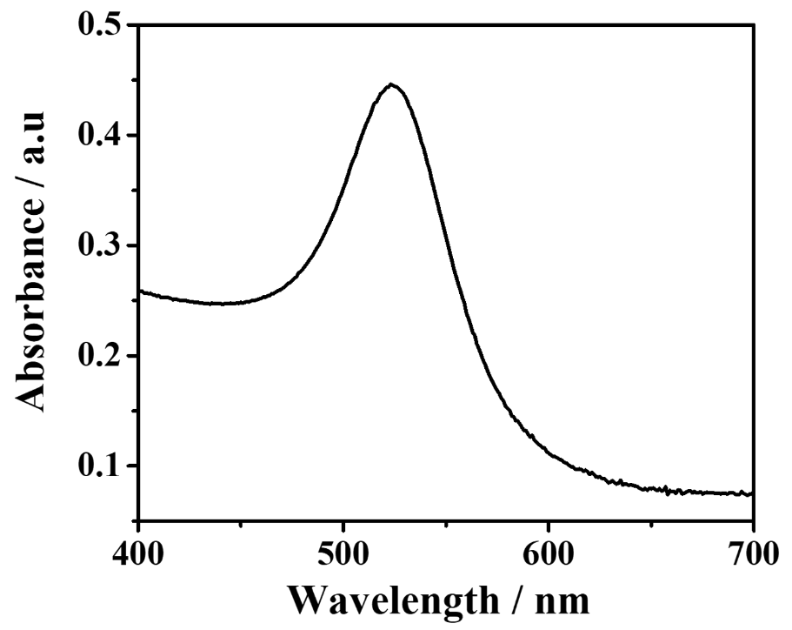


**Figure S1.** UV-Visible absorption spectrum of PEI-AuNPs solution synthesized in this study. The absorption spectrum of PEI-AuNPs solution exhibits a maximum peak at 524 nm.


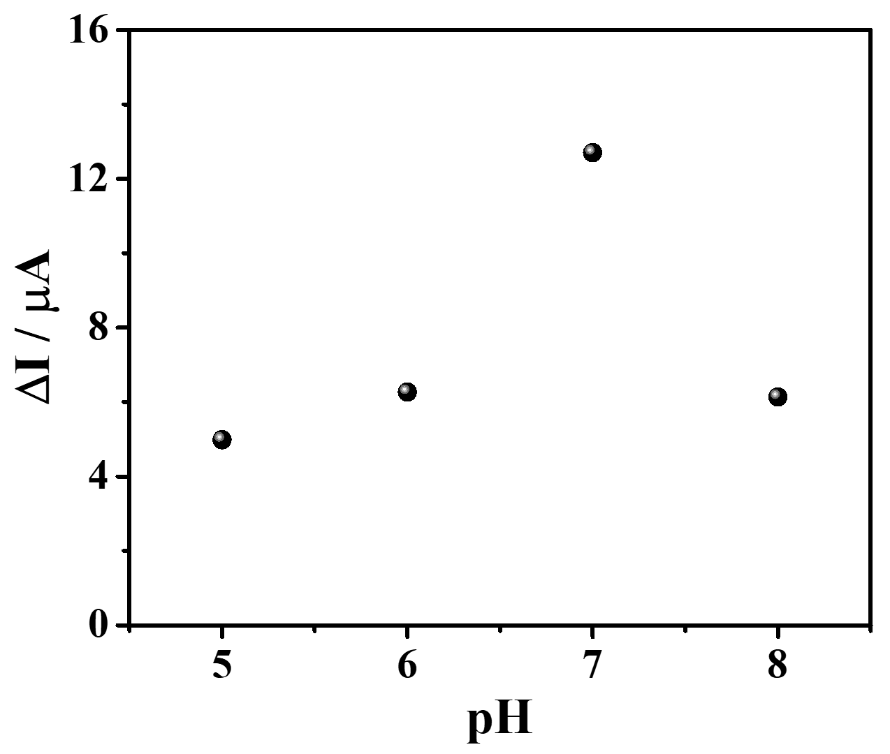


**Figure S2.** Effect of pH (0.010 M PB solution) on the detection based on the current change (ΔI) between before and after incubating with a solution containing AFP (50 ng mL^−1^).


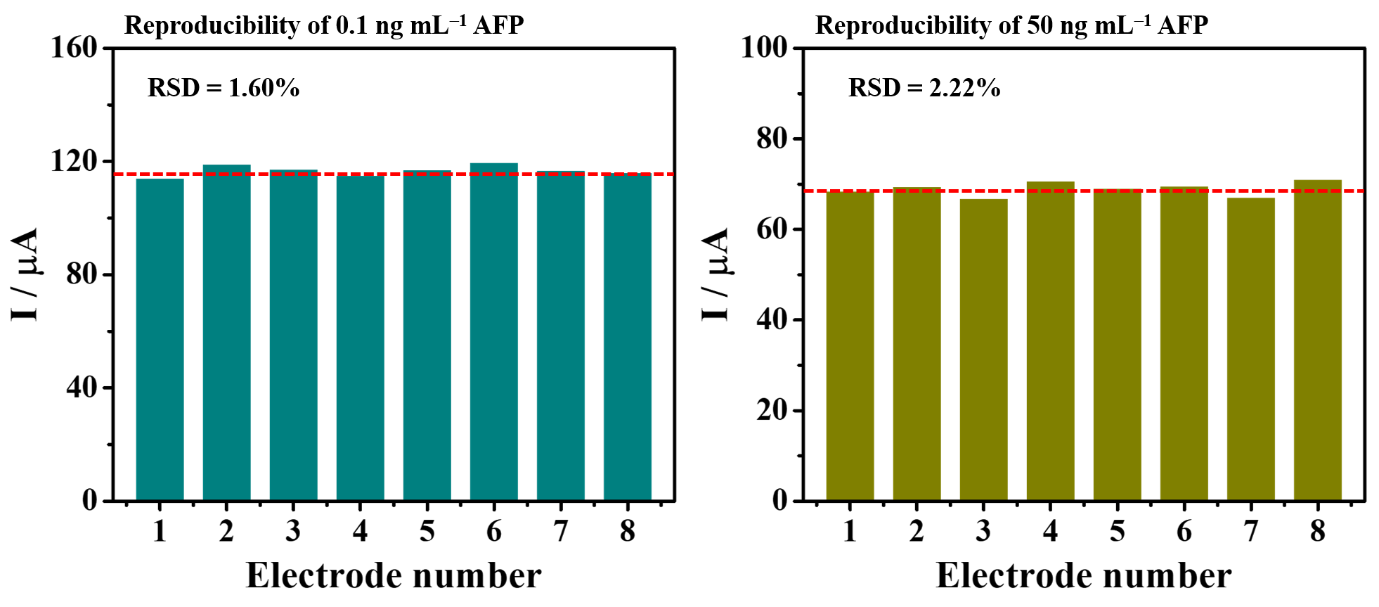


**Figure S3.** Reproducibility of the TB/BSA/Apt/PEI-AuNPs-based aptasensor, after binding with 0.10 and 50 ng mL^−1^ AFP

**Table S1** Comparison of a simple label-free electrochemical aptasensor and other reported methods for sensitive detection of AFP.

| **No.** | **Materials and modified electrode** | **Technique** | **Type** | **Linear range**  **(ng mL^−1^)** | **LOD**  **(ng mL^−1^)** | **Refs.** |
| --- | --- | --- | --- | --- | --- | --- |
| 1 | hemin@ZIF-67 | chemiluminescence | - | 0.4−20 | 0.133 | Wang et al., (2022) |
| 2 | Apt-QD1/SNPs (receptor) and Ab-AuNPs (donor) | fluorescence | sandwich | 0.5−45 | 0.40 | Zhou et al., (2019) |
| 3 | Aptamer@Trigger + F@Q + H1 + H2 | fluorescence | - | 0.1−10000 | 0.033 | Li et al., (2021) |
| 4 | BSA/Apt/PtNPs/GO-COOH/SPGE | SWV | label-free | 3−30 | 1.22 | Upan et al., (2021) |
| 5 | BSA/Aptamer/GO/GCE | CV | label-free | 0.01−100 | 0.003 | Yang et al., (2018) |
| 6 | PBNP-Apt/GO/gold-disk electrode | DPV | enzyme-free | 0.01−300 | 0.0063 | Zhang et al., (2019) |
| 7 | AFP-Apt/PtNPs/RGO−CS−Fc/Au NPs/SPCE | DPV | label-free | 1−10000 | 0.3013 | Li et al., (2020) |
| 8 | AFP-aptamer/TH/RGO/AuNPs/SPE | DPV | label-free | 100−100000 | 50 | Li et al., (2018) |
| 9 | BSA/AFP-Ab/Pd−rGO/GCE | DPV | label-free | 0.01−12 | 0.005 | Qi et al., (2014) |
| 10 | TB/BSA/Apt/PEI-AuNPs/SPE | DPV | label-free | 0.01-50 | 0.0095 | This work |

Zeolitic imidazolate framework (ZIF-67); aptamer (Apt); quantum dots (QDs); SiO_2_ nanoparticles (SNPs); antibody (Ab); gold nanoparticles (AuNPs); (FAM-DNA and quenched group-BHQ1 (F@Q); hairpin 1 (H1); hairpin 2 (H2); bovine serum albumin (BSA); platinum nanoparticles (PtNPs); carboxylated-graphene oxide (GO-COOH); screen-printed graphene-carbon paste electrode (SPGE); glassy carbon electrode (GCE); prussian blue nanoparticle (PBNP); alpha-fetoprotein-aptamer (AFP-Apt); reduced graphene oxide−chitosan−ferrocene nanohybrids (RGO–CS–Fc); screen-printed carbon electrode (SPCE); thionin (TH); reduced graphene oxide (RGO); screen-printed electrode (SPE); palladium−reduced graphene oxide (Pd−rGO); toluidine blue (TB); polyethyleneimine-coated gold nanoparticles (PEI-AuNPs).
